# Numb-associated kinases regulate sandfly-borne Toscana virus entry

**DOI:** 10.1080/22221751.2024.2382237

**Published:** 2024-07-17

**Authors:** Yarden Moalem, Rodolfo Katz, Anand G. Subramaniam, Yehonathan Malis, Yakey Yaffe, Nofit Borenstein-Auerbach, Keshet Tadmor, Roey Raved, Ben M. Maoz, Ji Seung Yoo, Yaniv Lustig, Chen Luxenburg, Eran Perlson, Shirit Einav, Ella H. Sklan

**Affiliations:** a Department of Clinical Microbiology and Immunology, Faculty of Medical & Health Sciences, Tel Aviv University, Tel Aviv, Israel; b Department of Physiology and Pharmacology, Faculty of Medical & Health Sciences, Tel Aviv University, Tel Aviv, Israel; c Department of Pathology, Faculty of Medical & Health Sciences, Tel Aviv University, Tel Aviv, Israel; d The Drimmer-Fischler Family Stem Cell Core Laboratory for Regenerative Medicine, Tel Aviv University, Tel Aviv, Israel; e Department of Cell and Developmental Biology, Faculty of Medical & Health Sciences, Tel Aviv University, Tel Aviv, Israel; f Sagol School of Neuroscience, Tel Aviv University, Tel Aviv, Israel; g School of Neurobiology, Biochemistry and Biophysics, George S. Wise Faculty of Life Sciences, Tel Aviv University, Tel Aviv, Israel; h Department of Biomedical Engineering, Tel Aviv University, Tel Aviv, Israel; i School of Life Sciences, BK21 FOUR KNU Creative BioResearch Group, Kyungpook National University, Daegu, Republic of Korea; j Central Virology Laboratory, Ministry of Health, Chaim Sheba Medical Center, Tel Hashomer, Israel; k School of Public Health, School of Medicine, Tel Aviv University, Tel Aviv, Israel; l Department of Medicine, Division of Infectious Diseases and Geographic Medicine, Stanford University, Stanford, CA, USA; m Department of Microbiology and Immunology, Stanford University, Stanford, CA, USA; n Chan Zuckerberg Biohub, San Francisco, CA, USA

**Keywords:** Toscana virus, Sandfly, Phleboviruses, Bunyaviruses, viral entry

## Abstract

Sandfly-borne Toscana virus (TOSV) is an enveloped tri-segmented negative single-strand RNA *Phlebovirus*. It is an emerging virus predominantly endemic in southwestern Europe and Northern Africa. Although TOSV infection is typically asymptomatic or results in mild febrile disease, it is neurovirulent and ranks among the three most common causes of summer meningitis in certain regions. Despite this clinical significance, our understanding of the molecular aspects and host factors regulating *phlebovirus* infection is limited. This study characterized the early steps of TOSV infection. Our findings reveal that two members of the Numb-associated kinases family of Ser/Thr kinases, namely adaptor-associated kinase 1 (AAK1) and cyclin G-associated kinase (GAK), play a role in regulating the early stages of TOSV entry. FDA-approved inhibitors targeting these kinases demonstrated significant inhibition of TOSV infection. This study suggests that AAK1 and GAK represent druggable targets for inhibiting TOSV infection and, potentially, related *Phleboviruses*.

## Introduction

Sandfly-borne viruses (*Toscanaense, Siciliaense*, and *Napoliense*), transmitted by phlebotomine sandflies, are medically relevant viruses that cause a self-limiting febrile disease known as sandfly fever [1]. Several Sandfly virus outbreaks have been previously described, mainly in the Mediterranean. The sandfly vectors, however, are spreading to new geographic regions, and new species are repeatedly isolated, some of which are lethal in mouse models [2]. Sandfly-borne Toscana virus (TOSV) is the only currently known phlebovirus causing human central and peripheral nervous system infections [3]. TOSV was found to underlie 4% of meningoencephalitis cases between 2006 and 2016 in Southwest Germany [4].

Sandfly-borne viruses are single-strand tri-segmented negative-sense, enveloped RNA viruses from the *Phlebovirus* genus (order *Bunyavirales:* family *Phenuiviridae*). Owing to their tri-segmented genomes, *Bunyaviruses* are prone to genetic reassortment. It was suggested that most currently recognized bunyaviruses are reassortants [5]. Currently, there is no vaccine or specific treatment for these viruses. Ideally, for viruses with a high reassortment probability, such modalities should have a pan*-Phenuiviruses* or pan-*Bunyavirus* potential. A significant barrier to developing such modalities is these viruses’ poorly understood life cycle. Thus, to facilitate the development of effective antiviral strategies, there is an urgent need to better understand the life cycle of these viruses [6].

The entry of all tested *Phenuiviruses* appears to depend on endosomal pH, suggesting the utilization of endocytic pathways [7]. A recent study described TOSV late entry steps, highlighting an acid-activated membrane fusion and an atypical late penetration [8]. However, host factors regulating earlier binding and internalization steps remain largely uncharacterized. Viruses typically utilize host endocytic pathways to invade host cells. Clathrin-mediated endocytosis of viral particles is triggered by the binding of the viral glycoprotein to a host receptor. The virus particle-receptor complex triggers endocytosis by forming a coated pit on the plasma membrane, leading to endosome formation. The coated pits are formed by clathrin adaptors that mediate the interaction between clathrin and cargo. Adaptor protein 2 (AP2) is the main adaptor protein in the plasma membrane [9]. Its phosphorylation enhances cargo recruitment, vesicle assembly, and internalization. Here, we focused on two members of the numb-associated Ser/Thr kinases (NAK) family: adaptor-associated kinase 1 (AAK1) and cyclin G-associated kinase (GAK) known endocytosis regulators. These kinases were shown to phosphorylate AP2 on Threonine 156 [10, 11]. AAK1 and GAK have additional non-redundant roles in endocytosis and other pathways (reviewed in [12]). AAK1 and GAK were shown to mediate the entry of multiple viruses [13–19], highlighting them as potential broad-spectrum antiviral targets. Our results suggest that AAK1 and, to a lesser extent, GAK mediate TOSV entry. Approved pharmacological inhibitors of these proteins inhibit viral entry, further highlighting their potential pan*-Phenuiviruses* antivirals.

## Methods

### Cells, viruses, compounds, and plasmids

Huh7 (JCRB cell bank 0403), U-87 MG (ECACC, 89081402), Vero (ATCC, CCL-81), and HEK-293FT cells (Thermo Fisher, R70007) were maintained in Dulbecco’s modified Eagle’s medium (DMEM, Gibco) supplemented with 10% fetal bovine serum (FBS, Gibco),1% (v/v) penicillin/streptomycin, and 1% L-glutamine (Biological Industries) at 37°C, in a humidified incubator with 5% CO2. TOSV strain UVE/TOSV/1972/IT/ISS PHL3, lineage A (Ref-SKU:001v-EVA981), Sandfly virus Sicilian (SFSV, strain UVE/SFSV/1943/IT/Sabin, Ref-SKU:001v-EVA77), and Naples (SFNV, UVE/SFNV/UNK/IT/30451, Ref-SKU:001v-02386) were from the European Virus Archive. Viral stocks were prepared in Vero cells, and the supernatant was harvested 2–5 days post-infection. Viral titers were determined by plaque assay on Vero cells. Plasmids expressing AP2M1 and the T156A mutant were described [20]. The compounds used are listed in the supplementary methods.

### Infection assays

Huh7 cells were pretreated with DMSO or inhibitors. The cells were infected with TOSV at the indicated multiplicity of infection (MOI) in the presence of the inhibitors. Viability was determined the following day using PrestoBlue (Thermo Fisher), and qPCR was used to determine intracellular viral RNA levels. Extracellular virus levels were determined using plaque assays.

### Strand-specific RT-qPCR

The assay was designed to distinguish between the genome and antigenome strands of the L segment as described in Tercero et al. with modifications ([21], see Figure S3 and primers in Table 1). Three separate RT reactions and PCR were performed. For the genome strand reaction: cDNA synthesis was performed using a tagged RT primer containing a non-viral sequence at the 5′ and a sequence complementary to the genome RNA segment (G-RT). This primer will not bind the antigenome as it is in the same orientation. The non-viral sequence was added to reduce false priming. For the PCR reaction, the non-viral tag sequence of the RT primer was used as the forward primer (G-PCR-F), and a strand-specific primer was used as the reverse primer (G-PCR-R). For the antigenome reaction: A primer complementary to a sequence in the 3′ UTR of the antigenomic strand absent from the mRNA transcript was used for RT. For the PCR reaction, the RT primer was used as a forward primer, with an antigenome-specific reverse primer (AG-PCR-R). For the housekeeping control reaction: HPRT cDNA was prepared using random primers.

**Table 1.** Primers used in the Strand-specific RT-qPCR reactions. The non-viral sequence tag is in bold and underlined.

Primer name	Fw	Rev
AG-RT	5′ ACACCCCCCACACCTATTCTCTCCCTCAGT 3′	
AG-PCR-R	5′ GGCTCTAGACAGAGACCCATC 3′	
G-RT	5′ **GGCCGTCATGGTGGCGAATA**AATATGGAAAGAATCCTTAGAAAACAACC 3′	
G-PCR	5′-**GGCCGTCATGGTGGCGAATA**-3′	5′ TGCTTGATCACATAGGCTGGT 3′
HPRT	5′ TGACACTGGCAAAACAATGCA 3′	5′ GGTCCTTTTCACCAGCAAGCT 3′

### shRNA stable cell lines

shRNA lentiviral plasmids targeting AAK1 (TRCN0000001946) and GAK (TRCN0000002158), or non-targeting (SHC002) were from Sigma. Lentiviruses produced from these plasmids were used to infect Huh7 cells (see supplementary methods).

### Binding Assay

Huh7 cells were pretreated with 10 μM inhibitors, or DMSO, for 30 mins on ice. The cells were infected at MOI of 5 for one hour on ice. Treatment with Proteinase-K (QIAGEN, 0.25 mg/ml) during infection was used as a control to remove the bound virus. Viral RNA levels were determined using qPCR.

### Entry assay

Huh7 cells were pretreated with DMSO, 20 mM NH_4_Cl, or 10 μM inhibitors and infected at MOI of 5. After two hours, the cells were harvested, and viral RNA levels were determined using qPCR.

### Replication assay

U-87 cells were infected on ice for one hour and shifted to 37°C for additional 1.5 hours. The media was replaced with media containing DMSO, 10 μM of the inhibitors, or 100 μM ribavirin. Viability was determined after six hours. Viral replication was determined by measuring antigenome RNA levels using qPCR [21].

### Additional assays

The details of the Quantitative Real-Time Reverse Transcription PCR (qRT-PCR).

Western blot and Immunofluorescence assays and the preparation of human iPSC-derived neurons are described in the supplementary methods.

## Results

### Clathrin-mediated endocytosis has a role in TOSV infection.

**Figure 1.**
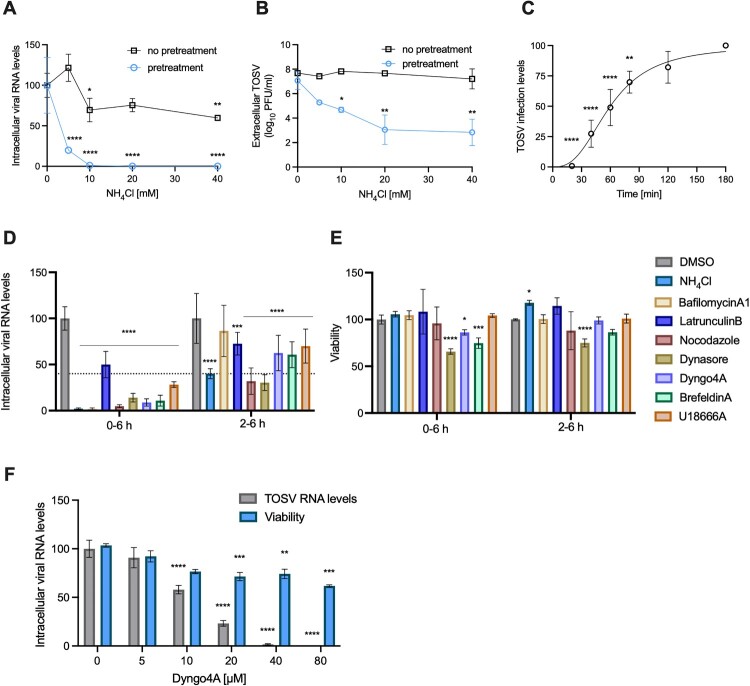
**Toscana virus entry depends on endosomal pH.** Huh7 cells were pretreated with the indicated concentrations of NH_4_Cl, followed by infection with TOSV (MOI = 1). **(A)** Intracellular viral RNA levels were measured after 24 hours. **(B)** Infectious virus production was determined using plaque assay. Data are plotted relative to DMSO control. **(C)** Huh7 cells were incubated on ice with TOSV (MOI = 1). After one hour, the cells were shifted to 37°C, and NH_4_Cl was added at the indicated time points. The cells were stained with a TOSV-specific antibody after 24 hours. Infection levels were calculated based on the fluorescence intensity. Infection levels after 180 min were regarded as 100% infection. **(D–E)** The effect of endocytosis affecting agents on TOSV entry and cell viability. Huh7 cells were infected with TOSV (MOI = 0.5) and treated with the indicated inhibitors during the infection (0–6 h) or two hours post-infection (2–6 h). Relative viability and intracellular viral RNA levels were determined six hours post-infection. Data are plotted relative to DMSO control. (**F**) Dose-response of dyngo-4A on TOSV RNA levels. RNA was extracted and analyzed 24 hours post-infection. Data in A and B are mean, ± standard deviation (SD) from a representative experiment out of three performed in triplicates. The results in C are mean ± SD from one experiment performed in triplicates. One-way ANOVA. Results in D and E are mean ± SD, and in F are mean ± standard error of the mean (SEM) from two independent experiments performed in triplicates. Two-way ANOVA, Dunnett’s multiple comparisons test, ****, *p* < 0.0001, ***, *p* < 0.001, **, *p* < 0.01, *, *p* < 0.05.

To determine the endocytic route used by TOSV and the experimental time frame, the weak base NH_4_Cl was used to confirm that entry is pH-dependent. Huh7 cells were pre-incubated with increasing amounts of NH_4_Cl, followed by infection with TOSV. NH_4_Cl inhibition was dose-dependent, reducing intracellular TOSV RNA levels to 19.8 ± 1.5% following treatment with 5 mM NH_4_Cl and to less than 1% when the cells were pretreated with 10 mM NH_4_Cl or more (Figure 1(A)). In agreement with these results, secreted TOSV levels were dramatically reduced by 4.36-logs when cells were pretreated with 40 mM NH_4_Cl (Figure 1(B)). Immunofluorescence confirmed the dose-dependent effect, with TOSV staining decreased to 4.7% at the lowest NH_4_Cl concentration tested (10 mM) and almost undetected levels (0.84%) at 40 mM (Figure S1A). Similar results were observed with the related SFNV and SFSV, where infection levels were reduced to 1.65% and 0%, respectively (Figure S1B and C).

We performed a time-of-addition experiment to identify the time frame at which TOSV fuses with the endosome. Huh7 cells were incubated with TOSV on ice for one hour to allow binding, then transferred to 37°C, and NH_4_Cl was added at different time points. Infection was detected using immunofluorescence (Figure 1(C) and raw data in S1D). Infection levels at each time point were quantified and used to calculate the time 50% of the virus entered the cells, which was 59.7 ± 3.3 min. Thus, our subsequent experiments used a two-hour time frame for infection. These results generally agree with a detailed analysis of the late stages of TOSV entry, establishing TOSV as a late penetrating virus [8].

To further identify the endocytic route involved in TOSV infection, we tested the effect of endocytosis affecting agents on infection. The agents were added either during the infection or two hours post-infection and kept present until cell lysis six hours post-infection. This time frame was selected as it represents a single cycle of infection. When added during infection, NH_4_Cl severely diminished viral RNA levels. Similar results were obtained when the cells were treated with bafilomycin, an inhibitor of endosome maturation, the inducer of microtubule depolymerization nocodazole, dynasore, a dynamin inhibitor that prevents clathrin-mediated endocytosis, dyngo-4A, a dynasore analog [22] and brefeldin A (BFA), an inhibitor of intracellular trafficking [23–25]. The actin cytoskeleton-altering drugs latrunculin B and U18666A, which inhibit lipid transport and late endosome maturation, had a milder effect on viral RNA levels ([26, 27], Figure 1(D)). When added two hours after infection, bafilomycin did not affect viral RNA levels. At the same time, latrunculin B, BFA, dyngo-4A, and U18666A had a mild effect, while nocodazole and dynasore had a more pronounced effect. All these effects were comparable to NH_4_Cl. Viability was not significantly affected by most of these drugs except for dynasore and BFA, which reduced viability by 25–35%. Thus, we tested the effect of different concentrations of the less toxic drug dyngo −4a. The drug inhibited TOSV RNA levels dose-dependently with a less pronounced effect on viability (14%, Figure 1(F)). These results suggest that clathrin-mediated endocytosis, intracellular trafficking, and microtubules play a role in TOSV entry.

### Knockdown of clathrin AP-2 regulators inhibit TOSV infection.

**Figure 2.**
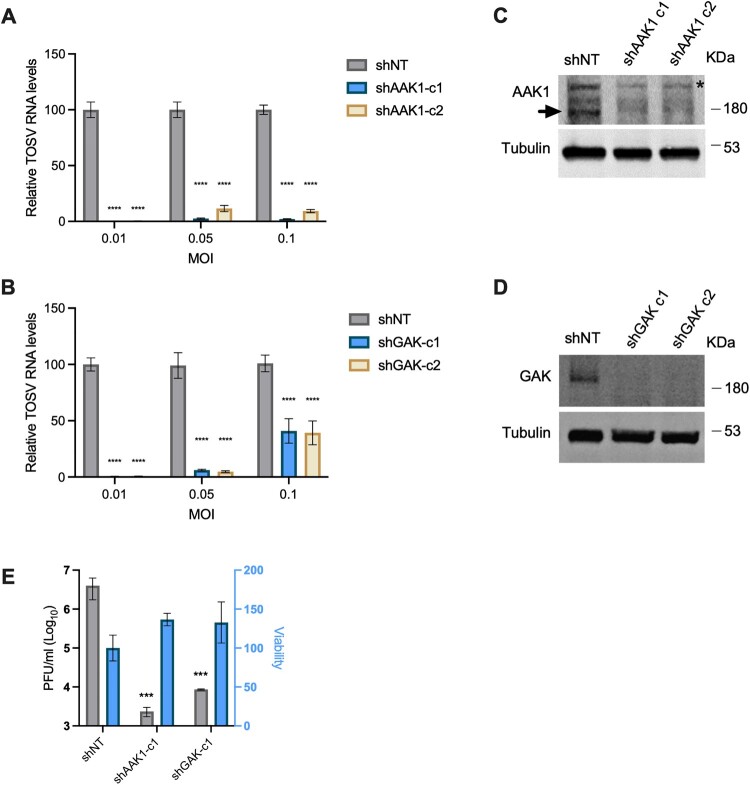
**Knockdown of AAK1 and GAK inhibits TOSV infection.** Huh7 clones stably expressing non-targeting (NT) shRNA, shAAK1 **(A)**, or shGAK **(B)** were infected with TOSV (MOI = 0.01, 0.05, or 0.1). RNA was extracted and analyzed 24 hours post-infection. Data are plotted relative to shNT control. **(C, D)** shRNA-mediated knockdown was confirmed by western blot using AAK1 and GAK-specific antibodies. Tubulin was used as a loading control. The position and size of the molecular weight markers (kDa) are indicated on the right. The arrowhead indicates the AAK1 band, and the asterisk indicates a non-specific band. **(E)** Huh7 clones stably expressing shNT, shAAK1, or shGAK were infected with TOSV (MOI = 0.1). After 24 hours, the media was collected to perform plaque assays on Vero cells. The results were normalized to cell viability. The results are mean ± SEM (A, B) or SD (E) from at least two independent experiments performed in duplicates or quadruplicates. Two-way ANOVA, Dunnett’s multiple comparisons test, ****, *p* < 0.0001, ***, *p* = 0.0009.

To identify host factors regulating TOSV entry before endosome fusion, we tested the role of AAK1 and GAK. Both kinases stimulate AP2 binding to cargo by phosphorylation, thus enhancing cargo recruitment, vesicle assembly, and internalization [10, 11]. Stable Huh7 lines expressing non-targeting (NT), AAK1, or GAK shRNAs were established. The cells were infected with TOSV and tested for viral RNA levels (Figure 2(A, B)). Knockdown was confirmed using Western blot (Figure 2(C, D)). AAK1 levels were reduced by an average of 53.3 ± 8.4%, while GAK levels were decreased by 97.2 ± 3.1% in both clones. Of note, infection seems to upregulate the levels of both kinases in Huh7 cells (Figure S2) due to the increased endocytosis or a viral regulatory mechanism. TOSV RNA levels were significantly decreased by 304.7, 14.2, and 17.4-folds on average in the shAAK1 cells infected with MOIs of 0.01, 0.05, and 0.1, respectively (Figure 2(A)) and by 154, 18.3 and 2.5-folds in the shGAK cells.

Interestingly, higher infection levels were measured in the shGAK cells when infected at a higher MOI. In contrast, a less pronounced elevation was observed under similar conditions with shAAK1 (Figure 2(B)). Similar results were obtained using plaque assay where shAAK1 reduced viral production by 3.2 logs and shGAK by 2.6 logs (Figure 2(E)). This finding indicates that AAK1 or a different pathway might compensate for the loss of GAK during infection with a higher virus inoculum. Overall, these results highlight a role for AAK1 and, to a lesser extent, GAK in TOSV infection.

### Pharmacological inhibition of TOSV infection.

To further confirm these results, we asked if TOSV infection could be inhibited with approved AAK1 and/or GAK inhibitors. Thus, we determined the effect of two compounds: sunitinib, an approved multi-kinase inhibitor with a potent AAK1 (dissociation constant (Kd) = 11 nM) and GAK binding activity (Kd = 20 nM), and erlotinib, which has potent epidermal growth factor receptor (EGFR, Kd = 0.7 nM) and GAK (Kd = 3 nM) binding activities [14, 28].

**Figure 3.**
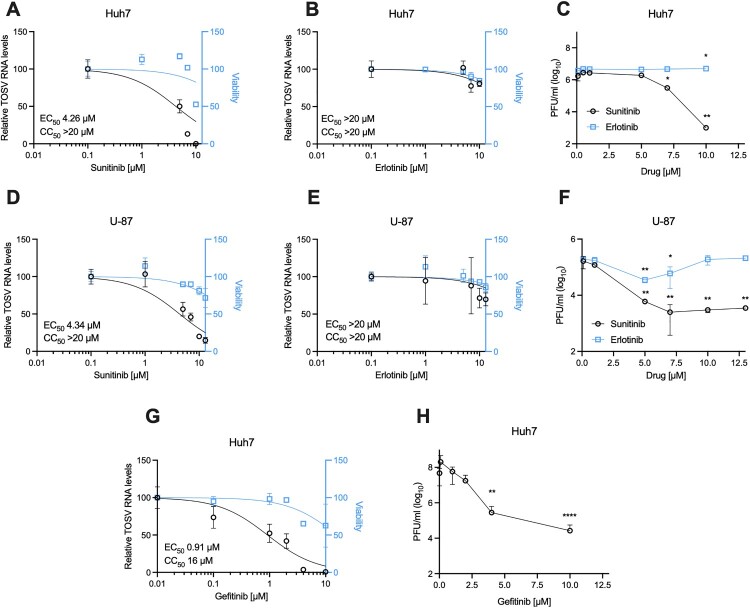
**Pharmacological inhibition of TOSV infection.** Huh7 cells were pretreated for 30 minutes with the indicated doses of **(A)** sunitinib or **(B)** erlotinib and infected with TOSV (MOI = 0.1). The drugs were present for the duration of the experiment. Viability (light blue) and TOSV RNA levels (black) were determined 24 hours post-infection. **(C)** Viral titer was determined using plaque assays. **(D–F)** Similar experiments were performed using U-87 MG cells (MOI = 0.05). **(G–H)** Huh7 cells were treated with gefitinib, followed by infection with TOSV (MOI = 0.1) and analysis as described above. The results are mean ± SD from at least two independent experiments performed in triplicates. Two-way ANOVA (C, F) or One-way ANOVA (H), Dunnett's multiple comparisons test, *, p<0.01, **, p<0.001, ****, p<0.0001.

Huh7 cells were pretreated with the indicated inhibitors and infected with TOSV, followed by analysis of viral RNA levels. Dose-dependent antiviral activity of sunitinib was observed, with a half-maximal effective concentration (EC_50_) of 4.26 μM and half-maximal cytotoxic concertation (CC_50_) > 20 μM. In contrast, erlotinib had no apparent effect (EC_50_ and CC_50_ values >20 μM, Figure 3(A, B)). In agreement, infectious virus production was reduced by 3.23 logs following sunitinib treatment. In contrast, erlotinib treatment did not show any significant effect (Figure 3(C)). Since TOSV invades the central nervous system, we also tested the impact of these drugs in cells isolated from a human glioblastoma patient (U-87 MG, Figure 3(D, E)). Similar results were observed in this cell line with an EC_50_ of 4.34 μM for sunitinib, a CC_50_ > 20 μM, and both EC_50_ and CC_50_ values above 20 μM for erlotinib. As expected, the amount of extracellular virus also decreased dose-dependently, with a 1.7 log reduction following sunitinib treatment and without a significant effect following erlotinib treatment (Figure 3(F)).

The absence of an antiviral effect of erlotinib against TOSV is unexpected, considering that GAK shRNAs successfully inhibited TOSV infection (Figure 2(B)). This might be explained by the fact that despite erlotinib’s potent GAK binding activity *in vitro*, its intracellular activity is less efficient (IC_50_ of 910 nM) [29, 30]. To address this point, we tested the effect of gefitinib, which has a potent GAK binding activity (Kd = 13 nM) and moderate intracellular anti-GAK activity (IC_50_ = 420 nM) [30]. Dose-dependent antiviral activity of gefitinib was observed, with an EC_50_ of 0.9 μM and CC_50_ > 20μM (Figure 3(G)). The amount of extracellular virus was reduced by 2.6 logs following gefitinib treatment (Figure 3(H)). These results suggest that pharmacological AAK1 and GAK inhibitors could efficiently inhibit TOSV infection.

### AAK1 and GAK inhibit TOSV entry.

**Figure 4.**
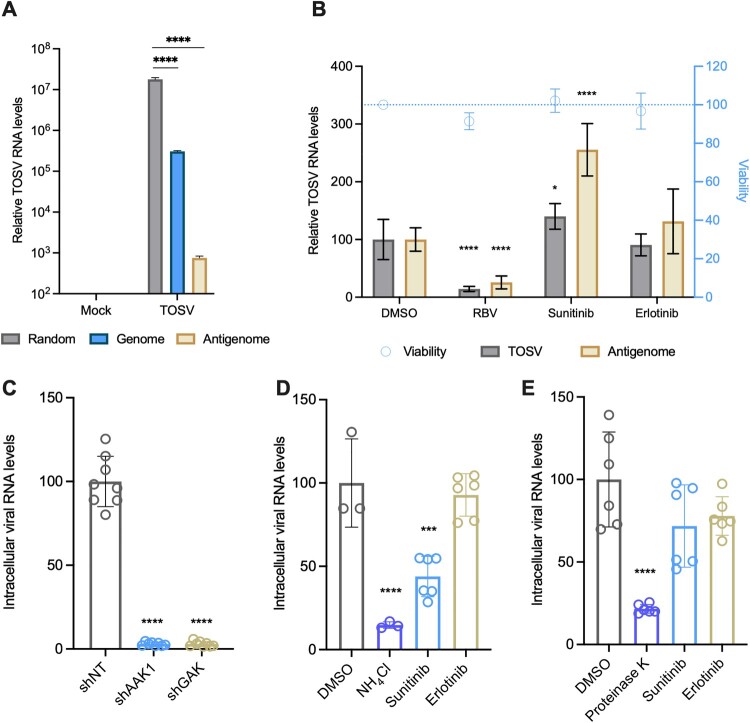
**AAK1 and GAK inhibit TOSV entry. (A)** Replication assay: U-87 MG cells were infected with TOSV (MOI = 1). The cells were harvested 24 hours post-infection and analyzed by strand-specific qRT-PCR. **(B)** U-87 MG cells were infected with TOSV (MOI = 0.5) on ice for one hour and then shifted to 37°C for an additional 1.5 hours. The media was replaced with media containing 10 μM of the indicated inhibitors. Viability was determined six hours post-infection (blue dashed line). The samples were analyzed as above. **(C)** Huh7 clones stably expressing shNT, shAAK1, or shGAK were infected with TOSV for two hours (MOI = 5). Viral RNA levels were determined following infection. **(D)** Huh7 cells were pretreated with the indicated inhibitors or NH_4_Cl. The cells were infected and analyzed as described in C. **(E)** Huh7 cells were pretreated with 10 μM of the indicated inhibitors for 30 minutes. The cells were then infected with TOSV (MOI = 1) for one hour on ice. Controls were treated with Proteinase K during infection. Viral RNA levels were determined following infection. Results are mean ± SD from two independent experiments performed in triplicates. **(A, B)** Two-way ANOVA, Dunnett’s multiple comparisons test, ****, *P* < 0.0001, *, *P* = 0.026. **(C–E)** One-way ANOVA. ****, *P* < 0.0001, ***, *P* = 0.0002.

To further understand if TOSV inhibition results from decreased virus binding, entry, or inhibition of a downstream step, we first tested the effect of sunitinib on TOSV replication. To distinguish between incoming viral RNA and viral replication, we quantified TOSV antigenome levels 6–8 hours post-infection in the presence of the inhibitors [21]. First, we validated the assay by quantifying the total amount of viral RNA and the amounts of genomic and antigenomic RNA in infected cells (Figure 4(A)). As expected, total viral RNA levels were higher than those of the genomic and antigenomic RNA, and genome levels were higher than antigenome levels, confirming the reliability of our assay. The assay was then performed in the presence of the inhibitors (Figure 4(B)). Ribavirin, a known replication inhibitor, was used as a control [31]. While ribavirin reduced viral replication by 78.7%, none of the other drugs affected viral replication. Cell viability was not affected by the inhibitors.

Next, we tested TOSV entry in shAAK1 and shGAK cells. The cells were infected at a high MOI for two hours. After infection, the cells were thoroughly washed, and internalized viral RNA levels were quantified. Viral entry was reduced by 97% in the shRNA-expressing cells compared to the non-targeting control (Figure 4(C)). Replication did not occur within this time frame as determined by antigenome quantification (Figure S4). To extend these results, TOSV entry was tested in the presence of sunitinib. Erlotinib was used as a negative control. Pretreatment with sunitinib decreased intracellular viral RNA levels by 56% compared to DMSO. Erlotinib pretreatment did not affect viral entry (Figure 4(D)). To confirm that the inhibitors didn’t affect viral binding, we tested virus binding after pretreatment with the inhibitors. Binding tests were conducted on ice to permit viral binding without fusion. Proteinase-K was previously shown to remove other bound viruses and, thus, was used as a control [32]. Pretreatment with either inhibitor did not significantly affect viral binding (Figure 4(E)). These results suggest that sunitinib and erlotinib do not interfere with TOSV replication and confirm that the inhibition occurs at a post-binding entry step.

### TOSV infection of iPSC-derived neurons.

**Figure 5.**
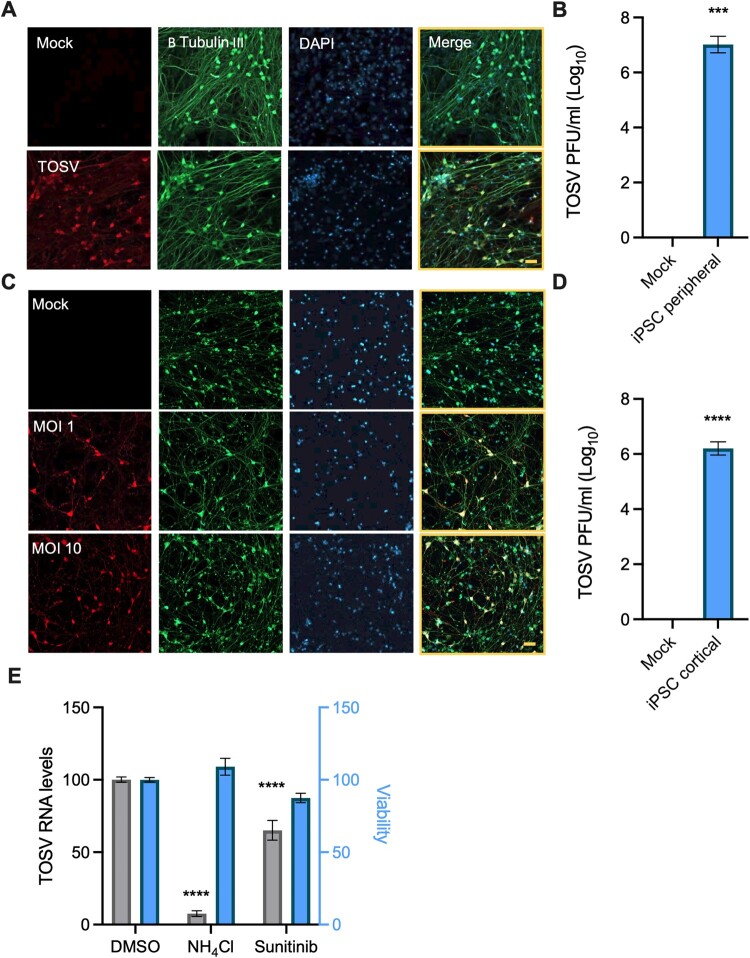
**TOSV infection of iPSC-derived neurons. (A)** iPSC-derived peripheral neurons from two healthy donors were infected with TOSV (MOI = 5) on day 24. The cells were fixed 24 hours post-infection and stained with TOSV (Red) and Class III β-Tubulin-specific antibodies (Green). DAPI staining is shown in blue. **(B)** Growth media from the experiment described in A was analyzed for extracellular virus levels. **(C)** iPSC-derived cortical neurons at day 14 after differentiation were infected with TOSV (MOI = 1 or 10). The cells were fixed and stained as described above. **(D)** Growth media was used for plaque assays **(E)** iPSC-derived mature cortical neurons on day 14 of the GENtoniK treatment were pretreated with sunitinib and infected with TOSV (MOI = 0.5). The cells were harvested 6 hours post-infection, and viability and viral RNA levels were determined. Results are mean ± SEM from two independent experiments performed in triplicates. Two-way ANOVA, Dunnett’s multiple comparisons test, ****, *P* < 0.0001, ***, *P* < 0.001, **, *P* < 0.01. Scale bars: 50 µm.

Severe TOSV infection can cause neurological manifestations [33–38]. Thus, we examined TOSV infection in iPSC-derived neurons. First, TOSV infection of peripheral neurons from two healthy donors was tested. Figure 5(A) shows infection levels in peripheral neurons from one donor after 24 hours. Similar results were obtained with neurons from the other donor. These neurons also produced infectious viruses, with extracellular virus levels reaching 10^7 ^pfu/ml 24 hours post-infection (Figure 5(B)). Next, we infected cortical glutamatergic neurons with TOSV. Infection levels were approximately 60% at MOI of 1 and 90% at MOI of 10 after 24 hours (Figure 5(C), in agreement with [8]), with extracellular virus levels reaching 10^6^ pfu/ml 24 hours post-infection (Figure 5(D)). These results demonstrate that TOSV effectively infects peripheral and cortical glutamatergic iPSC-derived neurons.

Neurons have specialized roles in synaptic vesicle release and retrieval. While clathrin-mediated endocytosis seems to be a key mechanism in this process, little is known about it in neurons. In rat hippocampal neurons, down-regulation of clathrin or inhibition of dynamin blocked synaptic vesicle retrieval. Knockdown of AP-2, however, had minor effects on retrieval, possibly due to compensation by AP-1 [39]. If AAK1 and GAK play a role in AP-2 regulation in TOSV infection in neurons, they might be amenable to pharmacological inhibition by sunitinib. To answer this question, neurons were pretreated with sunitinib and infected with TOSV for 24 hours. Sunitinib pretreatment caused a decrease in TOSV infection (EC_50_ of 3.76 μM, Figure S5). However, cytotoxicity was significantly higher (CC_50_ of 5.65 μM). The levels of extracellular virus dropped by 1–2.3 logs in this experiment (Figure S5). To reflect natural TOSV infection more accurately, iPSC-derived neurons were treated with the GENtoniK cocktail, driving neuron maturation [40]. These neurons were pretreated with sunitinib and infected with TOSV for 6 hours. Under these conditions, sunitinib significantly but moderately reduced TOSV infection without significantly affecting cell viability (Figure 5(E)). Suggesting AP-1 compensation or other mechanistic differences in endocytosis regulation might underlie the smaller effects of sunitinib in neurons.

### The effect of sunitinib on TOSV infection in mouse primary keratinocytes.

**Figure 6.**
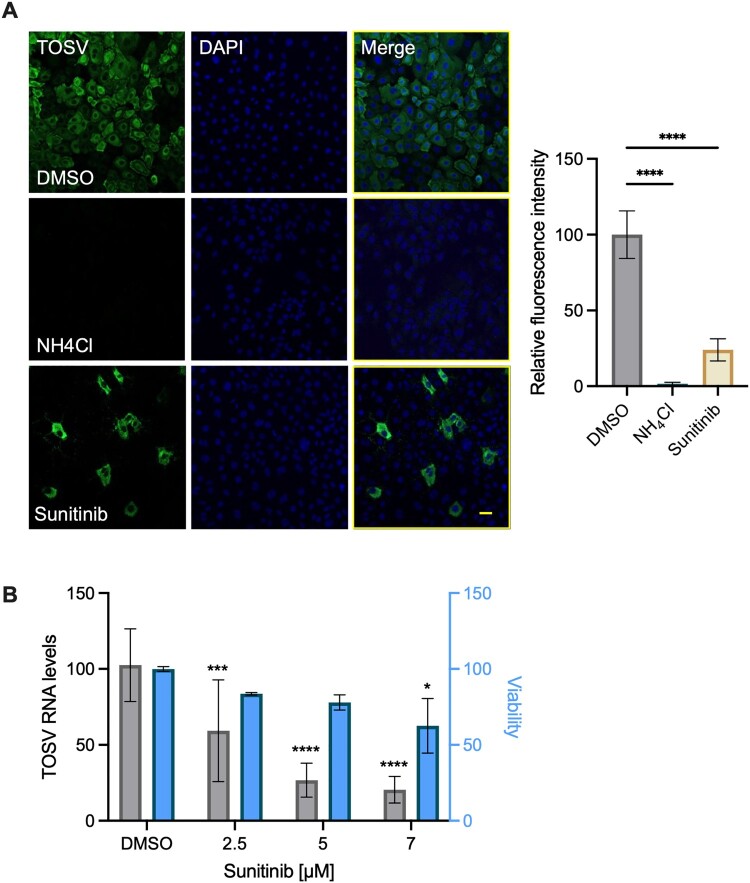
**Sunitinib inhibits TOSV infection in primary mouse keratinocytes. (A)** Primary mouse keratinocytes were pretreated with sunitinib (5 µM) or NH_4_Cl (40 mM), followed by infection with TOSV (MOI = 1). The cells were harvested 18 hours post-infection. The treatment continued for the duration of the experiment. The cells were fixed and stained with TOSV-specific antibodies (Green). DAPI staining is shown in blue. Scale bar = 50 µm. The graph shows the quantification of the fluorescent intensity from the images in the experiment. **(B)** Primary mouse keratinocytes were treated with the indicated concentrations of sunitinib and infected with TOSV (MOI = 0.05), as described above. After 18 hours, viability (light blue) and TOSV RNA levels (black) were determined. Results are mean ± SD from three images for each treatment (A) or two independent experiments performed in triplicates (B). One-way ANOVA (A), Two-way ANOVA (B), Dunnett’s multiple comparisons test, *, *p* < 0.05, ***, *p* < 0.001, ****, *p* < 0.0001.

The effect of sunitinib on TOSV infection in other primary cell types was tested in mouse primary keratinocytes. Keratinocytes were chosen as they are known to be the initial site of infection for many arboviruses [41, 42]. Immunofluorescence with a TOSV-specific antibody reveals efficient infection approaching 100% of these cells (Figure 6(A)). Treatment of the cells with sunitinib dramatically reduced TOSV infection levels, leaving only a small number of cells labelled with the TOSV antibody. While fluorescent intensity was reduced by 76% in the sunitinib-treated cells compared to the control. Under these conditions, viral production was reduced by 1 log, comparable to the 1.3 log reduction in the NH_4_Cl control. To further confirm these results, TOSV RNA levels were tested following sunitinib treatment (Figure 6(B)). Pretreatment with 5 µM sunitinib decreased intracellular viral RNA levels by 73.4% compared to DMSO without significantly affecting viability. A more pronounced effect was observed with 7 µM sunitinib, decreasing intracellular viral RNA levels by 79.8%. However, at this concentration, viability was reduced to 62%. These results suggest that TOSV efficiently infects primary mouse keratinocytes and that sunitinib can inhibit this infection.

### Inhibition of viral entry by sunitinib is mediated via AP-2 phosphorylation.

**Figure 7.**
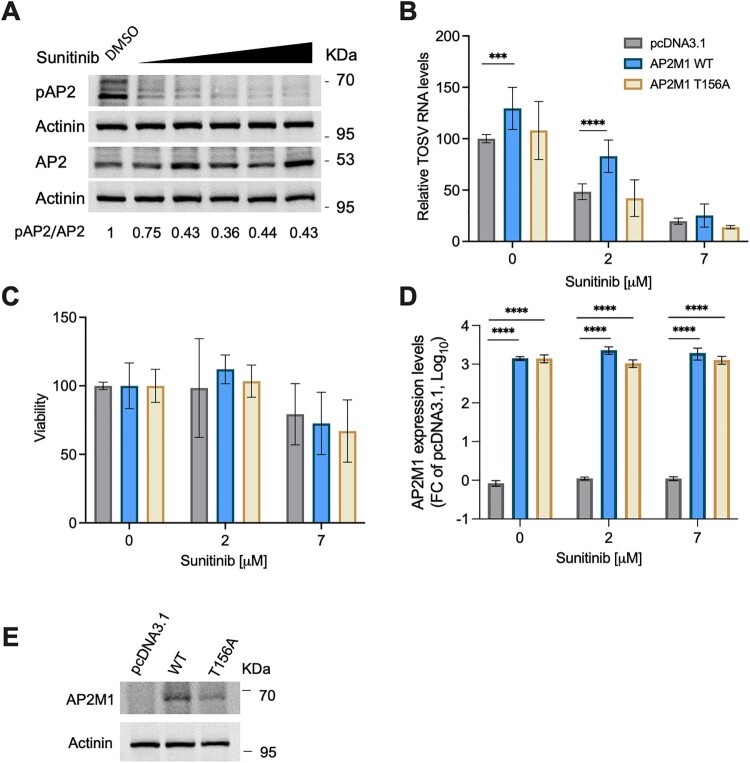
**Sunitinib inhibits TOSV entry by inhibiting AP2M1 phosphorylation. (A)** Huh7 cells were pretreated with sunitinib (1–10 μM) for 20 minutes and infected with TOSV (MOI = 2.5) for 30 minutes. The cell lysates were analyzed using western blot with the indicated antibodies. pAP2 levels normalized to actinin and total AP2 levels, expressed relative to the DMSO control, are shown. **(B)** Huh7 cells were transfected with an empty plasmid, AP2M1-expressing plasmid, or a T156A phosphorylation APM21 mutant. The cells were infected with TOSV (MOI = 0.05) the following day. The cells were harvested 24 hours post-infection, and viral RNA levels were determined. **(C)** Viability was determined 24 hours post-infection. **(D)** Expression levels of wild-type and mutant AP2M1 were determined using qPCR. **(E)** Western blot analysis was performed to confirm AP2M1 expression. Results are mean ± SD from two independent experiments performed in duplicates. Two-way ANOVA, Dunnett’s multiple comparisons tests, ***, *P* < 0.001, ****, *P* < 0.0001.

Sunitinib is a multi-kinase inhibitor with a potent AAK1 and GAK binding activity [28]. Phosphorylation of threonine 156 in AP2 subunit M1 by these kinases stimulates its binding to cargo proteins [10, 20, 43–45]. To identify the mechanistic details of sunitinib-mediated inhibition of TOSV entry, we probed the levels of phospho-AP2M1 following viral infection treatment. Phospho-AP2M1 levels decreased in sunitinib-treated infected cells in a dose-dependent manner (Figure 7(A)). Thus, to confirm that the inhibition is mediated through phospho-AP2M1 we tested if overexpression of AP2M1 could compensate for the lack of phosphorylation and rescue viral infection. A plasmid expressing a phospho-deficient AP2M1-T156A mutant was used as a control (Figure 7(B)). Overexpression of wild-type AP2M1, but not the phosphorylation mutant, significantly increased TOSV infection by ∼30%, suggesting that AP2 is rate-limiting for infection.

Furthermore, wild-type AP2M1 partially rescued viral infection when a low (2 μM) dose of sunitinib was used. This rescue was not observed upon overexpression of the phosphorylation mutant. Non-significant lower rescue levels were observed when a higher concentration of sunitinib was used (7 μM). Cell viability was not affected by the ectopic expression of AP2M1 (Figure 7(D)). The expression levels of wild-type and mutant AP2M1 were comparable as determined using qPCR (Figure 7(D)). Expression was confirmed using western blot as well (Figure 7(E)). These results suggest that sunitinib-mediated inhibition of AP2 phosphorylation underlies, at least in part, its anti-TOSV effect.

## Discussion

This study identified vital host factors, namely AAK1 and GAK, that are pivotal in regulating an early post-binding step during TOSV entry. Furthermore, we demonstrated that FDA-approved multi-kinase inhibitors effectively target these kinases and can inhibit infection. The effect of these drugs could be partially rescued by overexpression of AP2M1, suggesting that inhibition of AP2 phosphorylation underlies this antiviral effect. These results indicate that AAK1 and GAK regulate the internalization of TOSV.

This finding is significant as these inhibitors are among the first to demonstrate efficacy against sandfly-borne viruses. Furthermore, inhibition of AAK1 and GAK was shown previously to inhibit infection of many pathogenic viruses, highlighting them as potential targets for broad-spectrum antivirals [13–18]. While additional studies are needed to substantiate this assertion, AAK1, and GAK hold promise as potential broad-spectrum *Phleboviruses* or *Bunyavirus* targets. This approach is essential for this family of viruses, considering the substantial antigenic variability and high pathogenicity. As was previously shown for several other viruses, using AAK1 and GAK as drug targets will likely have a high barrier to resistance as these are host factors that are not under the genetic control of these viruses [13–18, 46].

We found that AAK1 is essential for TOSV infection while GAK appears to play a less critical role, as evidenced by the increased infection levels observed at a higher MOI in shGAK cells but not in shAAK1 cells (Figure 2) and the lack of effect of erlotinib (Figure 3).

Notably, the reduction of AAK1 and GAK demonstrates comparable impact on hepatitis C virus (HCV), Dengue virus (DENV), and severe acute respiratory syndrome coronavirus-2 (SARS-CoV-2) infections [13, 14, 16]. However, the influence on rabies virus differs, as GAK siRNA does not alter infection [18]. In addition, erlotinib exhibits variable efficacy in viral inhibition. Specifically, while erlotinib affects HCV and DENV infection, it does not significantly impact TOSV, SARS-CoV-2 (when administered alone), or rabies virus infections [13, 14, 16, 18]. Gefitinib, a drug with improved intracellular anti-GAK activity, showed moderate antiviral activity against SARS-CoV-2. This fact is explained by its improved biological activity against GAK [14]. This, however, might be different with TOSV and Rabies, where knockdown of AAK1 and GAK showed different results. One explanation for this difference is additional non-redundant functions attributed to these kinases [12, 47]. Thus, further studies addressing this point might reveal additional details about TOSV entry and the roles of AAK1 and GAK.

The differences in the inhibition efficiency of these multi-kinase inhibitors might also result from their different kinase inhibition profiles. Sunitinib is also a potent inhibitor of BMP-2 inducible kinase [28]. Furthermore, serine/threonine kinase 16, another NAK member, affects SARS-CoV-2 life cycle [14]. Thus, these NAKs might also be involved in TOSV infection.

Gefitinib and erlotinib are potent EGFR inhibitors [28]. While gefitinib activity on TOSV infection might be partially attributed to its effect on EGFR, several lines of evidence support a more central role of its GAK inhibitory effect. These include erlotinib’s lack of effect on TOSV infection, the shRNA experiments, and the correlation of sunitinib’s anti-viral activity with the reduction in pAP2 levels and the AP2 rescue experiments.

Given TOSV’s neurovirulent nature, we examined infection in iPSC-derived neurons. Our results expand a recent study showing that TOSV infects hiPSC-derived cortical neurons [8] by confirming that TOSV can generate infectious virus particles in both iPSC-derived cortical and peripheral neurons. While the clinical significance of TOSV infection in peripheral neurons remains uncertain, it is noteworthy that some patients exhibit symptoms linked to potential peripheral nervous system involvement [3]. Additionally, our findings raise the possibility that the peripheral nervous system could be the gateway for TOSV to access the central nervous system.

Interestingly, sunitinib had a lower but significant effect on infection in neurons. It is still unclear if these inhibition levels result from mechanistic differences in endocytosis regulation in these cells or other yet unknown mechanisms. In addition, our results show for the first time that TOSV can efficiently infect mouse primary keratinocytes. The fact that sunitinib could efficiently inhibit TOSV infection in mouse primary keratinocytes further supports this notion.

Interestingly, a combination of sunitinib and erlotinib suppressed systemic dengue virus infection in mice and delayed but did not prevent late-onset paralysis as these drugs are blood–brain barrier impermeable [17]. Similarly, sunitinib prolongs the survival of rabies-infected mice [18]. Thus, we hypothesize that sunitinib could similarly suppress TOSV systemic infection, lowering viremia and reducing the risk of central nervous system invasion in at-risk patients. Subcutaneous infection of mice with a neuroadapted TOSV strain leads to viremia and the detection of the virus in several tissues, including a very low titer in the brain (5 pfu/ml) without discernible symptoms [48]. Thus, this model is less suited to test this specific hypothesis.

Therefore, our study identified druggable host factors essential for TOSV infection for which no treatment is currently available. These targets represent a promising starting point for repurposing current drugs or developing new drugs against sandfly-borne virus infections.

## Supplementary Material

Supplementary material revised last.docx
