# Supplementary material for: Numb-associated kinases regulate sandfly-borne Toscana virus entry
Source: Emerg Microbes Infect. 2024 Jul 17;13(1):2382237. doi: 10.1080/22221751.2024.2382237 (PMC11285224; doi:10.1080/22221751.2024.2382237)
Supplement: Supplementary material revised last.docx [file TEMI_A_2382237_SM8650.docx]

**Supplementary figures:**

**
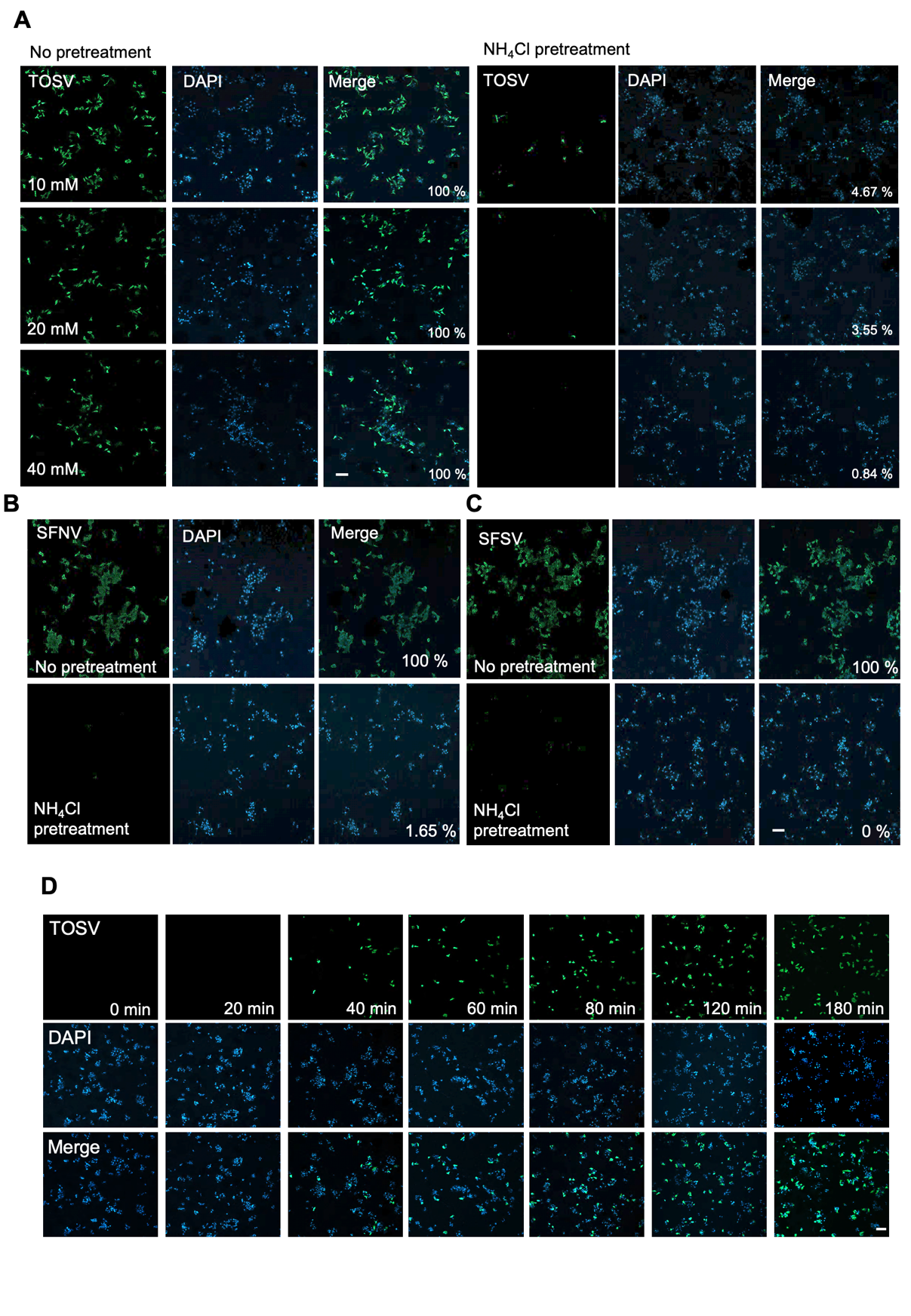
**

**
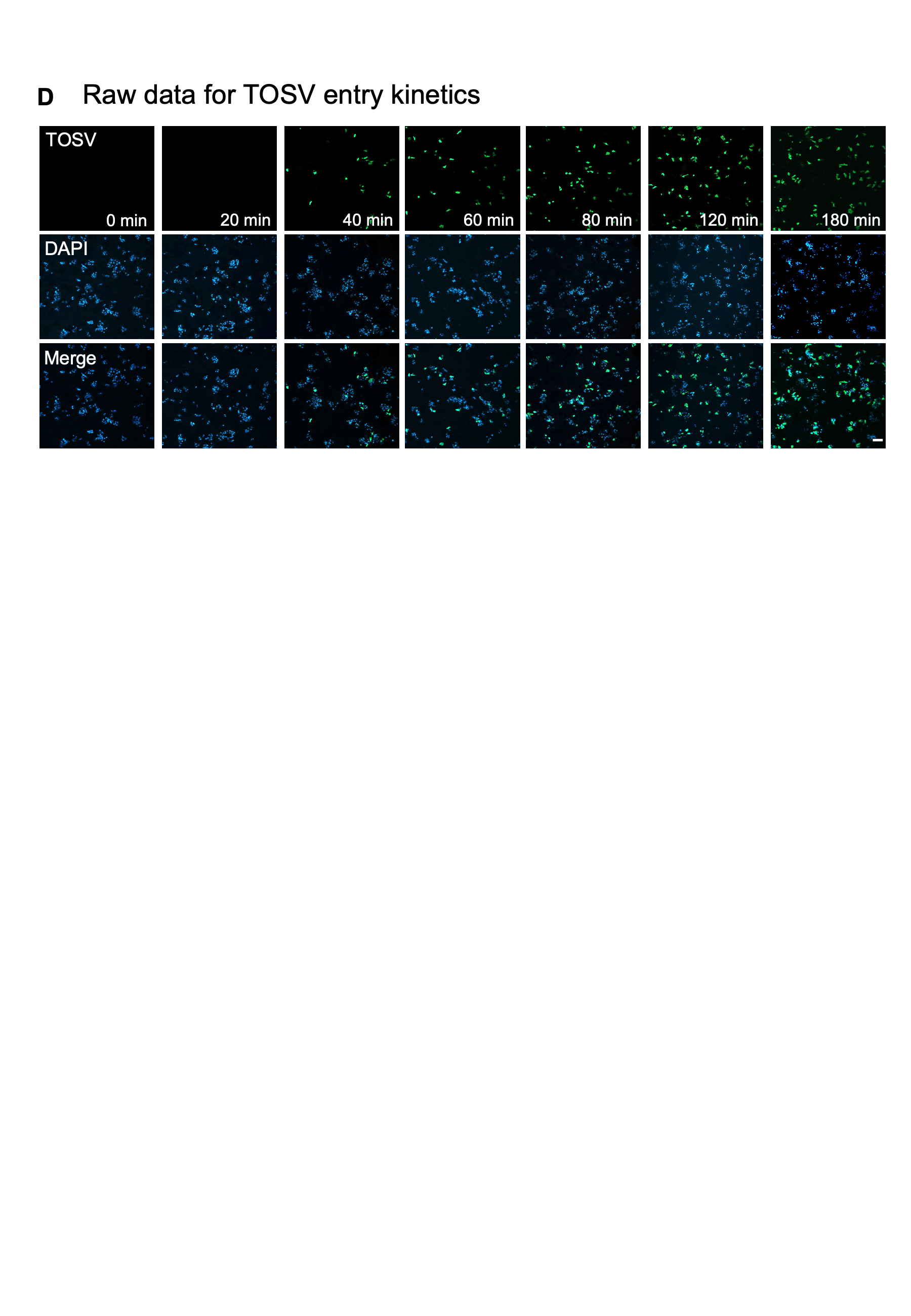
Figure S1: Sandfly virus entry depends on endosomal pH. (A)** Huh7 cells were pretreated with the indicated concentrations of NH_4_Cl for 30 min. The cells were infected with TOSV (MOI=1). Infection was detected after 24 hours using immunofluorescence. **(B-C)** Huh7 cells were pretreated with 40 mM of NH_4_Cl for 30 min and infected with SFNV and SFSV as described in A. Immunofluorescence was performed with virus-specific antibodies.

**(D)** Huh7 cells were incubated on ice with TOSV (MOI=1). After one hour, the cells were shifted to 37°C, and NH_4_Cl (40 mM) was added at the indicated time points. The cells were fixed after 24 hours and stained with a TOSV antibody. DAPI was used for nuclear staining in all the above experiments. All images were taken using a ZEISS LSM800 confocal microscope. Scale bar = 100 μm.

**
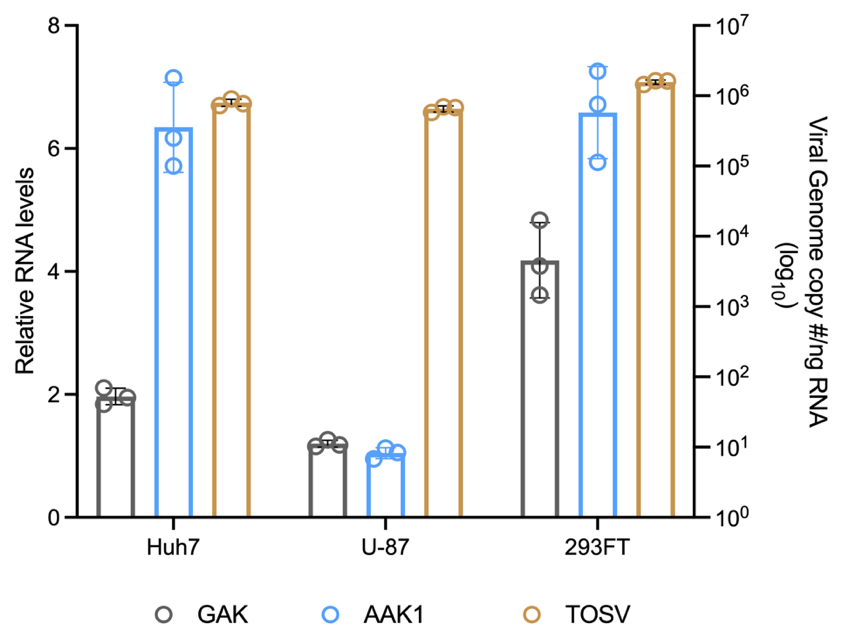
**

**Figure S2: TOSV infection affects AAK1 and GAK mRNA levels in different cell lines.** Huh7, HEK-293FT, and U-87 MG cells were infected with TOSV (MOI=1). RNA was extracted after 24 hours and analyzed using TOSV, GAK, or AAK1-specific primers. Viral genome copies were quantified using a standard curve. Results are mean ± standard deviation from one representative experiment out of three independent experiments performed in triplicates.

**
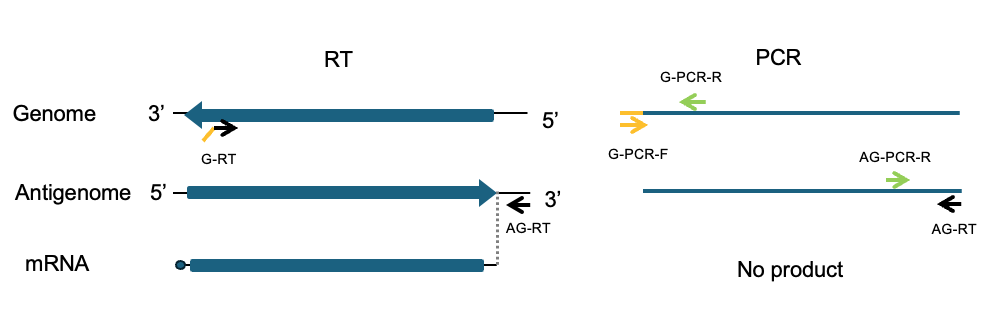
**

**Figure S3: Strand-specific RT-qPCR strategy.** A schematic drawing of the L-segment RNA species of TOSV is shown. RT primers are labeled in black, and the non-viral sequence is labeled in orange (left). The dashed line indicates the 3’-end of the full-length antigenome segment compared to the mRNA. PCR primers for viral sequences are labeled green, and the non-viral sequence in orange (right). Primer names corresponding to the names and sequences in Table S1 are shown. Adapted from Tercero et al. [1] with permission.

**Figure S4: Antigenome quantification two hours post-infection.** Huh7 cells were infected with TOSV for two hours (MOI=5). Viral antigenome levels were determined following infection using strand-specific RT-PCR as described in the methods section. Two-way ANOVA, Šídák's multiple comparisons test, ***, p=0.0003.

|  |
| --- |

**Figure S5: The effect of sunitinib on TOSV infection in iPSC-derived neurons.** iPSC-derived cortical neurons on days 14 or 15 were pretreated with sunitinib and infected with TOSV (MOI=0.01). The cells were harvested 24 hours post-infection, and viability and viral RNA levels were determined (Left). Plaque assays were performed with the growth media (Right). Two-way ANOVA, Dunnett's multiple comparisons test, **, p<0.01,***, p< 0.001,****, p<0.0001.

**Supplementary Methods**

*Quantitative Real-Time Reverse Transcription PCR (qRT-PCR)*

Total RNA was extracted using a Direct-zol or Quick-RNA kit (Zymo Research). RNA was reverse transcribed using an iScript cDNA synthesis kit (Bio-rad). Real-time PCR was performed using Fast SYBR green mix (ABI) and the primers listed in Table S1. TOSV primers targeted the NSs protein. Housekeeping controls were Hypoxanthine phosphoribosyltransferase 1 (HPRT) or Glyceraldehyde 3-phosphate dehydrogenase (GAPDH).

| Primer name | Fw | Rev |
| --- | --- | --- |
| TOSV | 5’ TCTCCCAGGAAATGACATCC 3’ | 5’ AGATGGGWGTCTCTGGTCAT 3’ |
| GAK | 5’ GTGGAGGAAGAGATCACGAGG 3’ | 5’ AGATATCCTGCTTCTCGCCG 3’ |
| AAK1 | 5’ CCACAAACTGAGGGAGTCAATGC 3’ | 5’ ATGTCTGCCTTCGTAGTGATGATTTT 3’ |
| AP2M1 | 5’ ACGTTAAGCGGTCCAACATTT 3’ | 5’ GCCATCACGTCACACATCTTAT 3’ |
| HPRT | 5’ TGACACTGGCAAAACAATGCA 3’ | 5’ GGTCCTTTTCACCAGCAAGCT 3’ |
| GAPDH | 5’ TGCACCACCAACTGCTTAGC 3’ | 5’ GGCATGGACTGTGGTCATGAG 3’ |

**Supplementary Table 1:** Primers used in the PCR reactions.

*Plaque assay*

Vero cells were infected with TOSV at 37°C for two hours and overlaid with a modified DMEM containing 5% methylcellulose (Sigma). After 9-11 days of incubation, the cells were fixed and stained with 0.1% crystal violet in 80% ethanol.

*Immunofluorescence assays*

Cells grown on coverslips were fixed with 4% paraformaldehyde and permeabilized using ice-cold 100% methanol. Blocking was with a 5% FBS and 0.3% Triton X-100 solution in PBS. The cells were incubated overnight with primary antibodies listed in Table S2. Secondary antibodies were Alexa Fluor, 488 goat anti-mouse, and 555 goat anti-rabbit (Invitrogen). Cell nuclei were stained with 4-6’ diamidino- 2 phenylindole (DAPI, Sigma). The cells were visualized using a confocal laser microscope (LSM800, Carl Zeiss).

*Compounds*

The following compounds were used. Final concentrations are listed in parenthesis for each drug where the concentration is not mentioned in the figure legend or body, and all the drugs were diluted in DMSO: sunitinib, erlotinib (Abcam), Gefitinib (Cayman), and Latrunculin B ((1 µM, Enzo Life Sciences). Brefeldin A (1 µM), Nocodazole (1 µM), Ribavirin (100 μM), and NH_4_Cl were from Sigma. Dynasore (60 µM), Dyngo-4a (40 µM), Bafilomycin A1(125 nM), and U-18666A (20 µM) were from the Cayman Chemical Company.

*Western blot*

Cells were lysed with radioimmunoprecipitation (RIPA) buffer (50 mM Tris-HCl pH 7.4, 150 mM NaCl, 1% Triton x-100, 1% Sodium deoxycholate, 0.1% SDS, 1 mM EDTA) supplemented with protease inhibitors (Sigma). For AP2M1 detection, calyculin A, a phosphatase inhibitor (Alomone labs), was added before cell lysis. Clarified lysates were loaded onto 10% SDS- polyacrylamide gels. The gel was transferred to a nitrocellulose membrane (Amersham). Membranes were blocked and blotted with the primary antibodies listed in Table S2. Signal was detected with secondary antibodies conjugated with horseradish peroxidase using enhanced chemiluminescence.

*Antibodies*

List of antibodies used for western blots and immunofluorescence (Table S1). Mouse anti-TOSV, SFNV, or SFSV antibodies (1:150, a gift from Prof. Jonas Schmidt-Chanasit, Bernhard Nocht Institute for Tropical Medicine, Hamburg).

|  | **Host** | **Application** | **Dilution** | **Company** | **Catalog #** |
| --- | --- | --- | --- | --- | --- |
| AAK1 | Rabbit | Western blot | 1:1,000 | Abcam | ab134971 |
| GAK | rabbit | Western blot | 1:1,000 | Abcam | ab305101 |
| AP2M1 | Rabbit | Western blot | 1:1,000 | Cell signaling | 68196S |
| phosphoAP2M1 | Rabbit | Western blot | 1:1,000 | Cell signaling | 3843S |
| α-actinin | Mouse | Western blot | 1:1,000 | Santa Cruz | sc-17829 |
| α-Tubulin | Mouse | Western blot | 1:10,000 | Sigma | T6199 |
| Class III β-Tubulin | Rabbit | Immunofluorescence | 1:350 | Sigma | T2200 |

**Supplementary Table 1:** List of primary antibodies for western blots and immunofluorescence.

*Lentivirus production*

The shRNA lentiviral plasmids were transfected to HEK293FT cells with the packaging plasmids pMDL-RRE pVSVG and pRev (Addgene) using polyethyleneimine (Polysciences Cat # 24765-2**).** After 48 h post-transfection, the media was collected and used to infect Huh7 cells by spinoculation (1000 g, 37°C, 45 minutes) in the presence of 4 µg/ml polybrene (Sigma). The following day, cells were transferred to a 10 cm plate supplemented with 0.75 µg/ml puromycin (Invivogen). The media and antibiotics were replaced every 2-3 days for about one week.

*Preparation of Human iPSC-derived neurons*

*Sympathetic neurons* were prepared at the Tel Aviv University Drimmer-Fischler Family Stem Cell Core Laboratory for Regenerative Medicine using a modification of the protocol described in Wu et al. [2]. Briefly, hPSCs (Sheba Medical Center Review Board approval #SMC-6158-19) were dissociated and mixed in differentiation medium containing Essential 6 (E6) medium (Gibco), bone morphogenetic protein 4 (0.4 ng/ml, Prospec), SB431542 (10 μM, Sigma) and CHIR99021 (300 nM, Sigma), and plated on Matrigel-coated plates for two days. The media was changed to a differentiation medium (days 2–10) containing E6 medium, 10 μM SB431542, and 0.75 μM CHIR99021 for neural crest cell induction. After 10 days, the aggregated spheroids were dissociated and resuspended on ultra-low attachment plates in spheroid medium (day 10–14) containing neurobasal medium, B27, Glutamax, N2 supplement (all from Gibco), 3 μM CHIR99021 and 10 ng/ml Fibroblast growth factor 2 (Axol bioscience). On day 14, sympathetic neuroblast spheroids were dissociated by accutase and plated on Poly-L-ornithine /laminin (Sigma)/fibronectin (Corning) coated plates in postganglionic sympathetic neuron (symNs) maturation medium containing neurobasal medium, B27, Glutamax, Glial-derived neurotrophic factor (25 ng/ml, PeproTech), Brain-derived Neurotrophic Factor (25 ng/ml, PeproTech), NGF (25 ng/ml, R&D systems), ascorbic acid (200 μM, Sigma), dbcAMP (0.2 mM, Sigma) and retinoic acid (0.125 μM, Sigma). The medium was changed every three days until day 20.

*Cortical glutamatergic neurons* were prepared using a modification of the Ward lab protocol [3]. Briefly, human iPSCs from the reference KOLF2.1J line were differentiated to stably express inducible human neurogenin 2 (NGN2) as previously described. iPSCs were differentiated into excitatory glutamatergic neurons with 2 mg/mL doxycycline (Sigma) for 72 h. Neurons were dissociated with accutase and plated at a density of 1.5x10^6^ cells per 6 well coated with poly-L-ornithine. iPSC-derived neurons were cultured for 14 days in BrainPhys Neuronal Medium (StemCell) supplemented with 2% B27 (GIBCO), 10 ng/mL BDNF, 10 ng/mL NT-3 (PeproTech), and 1 mg/mL mouse laminin (Corning). Half of the media volume was changed every 2–3 days for neuronal maintenance.

*Matured excitatory cortical neurons.* Cortical neurons were generated using a protocol based on the previously described dual-SMAD inhibition paradigm, followed by maturation driving treatment with GENtoniK cocktail (defined as a mixture of four small molecules – GSK2879552, EPZ- 5676, Bay K 8644, and NMDA, all from Cayman chemical) applied at a working concentration of 1 μM each and began 7 d after plating and maintained for 14 d [4]. Briefly, hiPSCs were dissociated into single cells with Accutase and plated at the density of 250,000/cm−2 onto Matrigel-coated plates in Nutristem medium with 10 μM Y-27632 (Tocris). During days 1–10 of the protocol, the medium consisted of Essential 6 (Thermo Fisher Scientific) with 10 μM SB431542 (Tocris) and 100 nM LDN193189 (Stemgent). Wnt inhibitor XAV-939 (Tocris) at 2 μM was included from day 1 to day 3 to improve anterior patterning. On days 11–20, the medium consisted of N-2-supplemented Dulbecco’s modified Eagle’s medium and Ham’s F-12 nutrient mixture (DMEM/F12; Thermo Fisher Scientific). Cells received daily medium exchanges throughout the differentiation. On day 20, cells were dissociated in Accutase for 30 min and plated on poly(l-ornithine) and laminin-coated (PLO/ Lam) plates in low-glucose (5 mM) Neurobasal A medium supplemented with 2% B27 and 1% GlutaMAX (Thermo Fisher Scientific) or cryopreserved in NutriFreez cryopreservation medium (Sartorius). Neurons received medium exchanges twice a week. During the first 7 d after plating, the medium was supplemented with notch-inhibitor N- (N-(3,5-difluorophenacetyl)-l-alanyl)-s-phenylglycinet-butyl ester (DAPT, Cayman Chemical), a γ-secretase inhibitor at 10 μM. Long-term cultures were maintained with brain-derived neurotrophic factor (BDNF, 10 ng/ml, PeproTech), glial cell line-derived neurotrophic factor (GDNF; 10 ng ml−1, R&D Biosystems), dibutyryl cAMP (100 μM, Sigma-Aldrich) and ascorbic acid (100 μM, Sigma-Aldrich). Treatment with the GENtoniK cocktail was applied at a working concentration of 1 μM each and began on day 7 after plating and maintained for 14 days.  *Mouse primary keratinocyte isolation* was performed as described in Nowak, J. A [5]. Briefly, dorsal skin was removed from newborn mice and incubated with dispase (Sigma-Aldrich). The epidermis was isolated and treated with trypsin (Biological Industries). Dissociated keratinocytes were plated on fibroblast feeder cells for four passages and then plated in tissue culture dishes without feeder cells.

*Statistical analysis*

Data were analyzed using GraphPad Prism 10 software and are presented as the mean with standard deviation or standard error as indicated. Analysis of variance (ANOVA) was performed when appropriate to assess the significance of variations. P values are as shown in the figure legend.

**References**

1. Tercero B, Terasaki K, Nakagawa K, et al. A strand-specific real-time quantitative RT-PCR assay for distinguishing the genomic and antigenomic RNAs of Rift Valley fever phlebovirus. J Virol Methods. 2019;272:113701.

2. Wu HF, Zeltner N. Efficient Differentiation of Postganglionic Sympathetic Neurons using Human Pluripotent Stem Cells under Feeder-free and Chemically Defined Culture Conditions. J Vis Exp. 2020 (159):e60843.

3. Fernandopulle MS, Prestil R, Grunseich C, et al. Transcription Factor-Mediated Differentiation of Human iPSCs into Neurons. Curr Protoc Cell Biol. 2018 Jun;79(1):e51.

4. Hergenreder E, Minotti AP, Zorina Y, et al. Combined small-molecule treatment accelerates maturation of human pluripotent stem cell-derived neurons. Nature Biotechnology. 2024 2024/01/02.

5. Nowak JA, Fuchs E. Isolation and Culture of Epithelial Stem Cells. In: Audet J, Stanford WL, editors. Stem Cells in Regenerative Medicine. Totowa, NJ: Humana Press; 2009. p. 215-232.
